# Identifying characteristic miRNAs-genes and risk pathways of multiple sclerosis based on bioinformatics analysis

**DOI:** 10.18632/oncotarget.23866

**Published:** 2018-01-02

**Authors:** Deling Luo, Jin Fu

**Affiliations:** ^1^ Department of Neurology, The Second Affiliated Hospital of Harbin Medical University, Nangang District, Harbin 150086, China

**Keywords:** miRNAs, multiple sclerosis, peripheral blood mono-nuclear cells, susceptibility gene, therapy

## Abstract

Multiple sclerosis is a chronic autoimmune disorder of the central nervous system. In MS, the genetic susceptibility is high and currently there is no effective treatment. MicroRNA, a small non-coding RNA, plays a vital role in immune responses. Aberrant or dysfunctional miRNAs may cause several diseases, including MS, thus miRNAs and miRNA related genes may be therapeutic weapons against MS. Here, we identified 21 miRNAs in peripheral blood mono-nuclear cells from over 600 persons, including healthy controls. By using informatics databases, 1637 susceptibility genes were evaluated and Cytoscape was used to integrate and visualize the relation between the miRNA identified and susceptibility genes. By using the cluster Profile package, a total of 10 risk pathways were discovered. Top pathways included: hsa05200 (pathway in cancer), hsa04010 (MAPK signaling pathway), and hsa04060 (cytokine-cytokine receptor interaction). By using the STRING database, a protein-protein interaction network was conducted to identify highly susceptibility genes. Moreover, the GSE21942 dataset was used to indicate the gene expression profiles and to correct prediction results, thereby identifying the most pivotal genes. The MiRSystem database provided information on both pivotal miRNAs and genes. In conclusion, miR-199a and miR-142-3p may be crucial for MS by targeting pivotal susceptibility genes, in particular *KRAS* and *IL7R.*

## INTRODUCTION

Multiple sclerosis (MS) is a chronic inflammatory neurological disorder in which auto-reactive immune cells attack the myelin sheath of nerve cell axons [1]. MS has higher incidence in females compared to males and affects young females between 20-40 years. Worldwide, nearly 2.5 million people suffer from MS [2]. A growing body of evidence shows that MS is associated with genetic susceptibility, environmental factors, virus infection, and autoimmune responses in which genes play a role that are closely related to MS [3, 4]. However, neither the underlying mechanism action nor the etiology of MS is clear and there is still no effective cure for the disease.

MicroRNAs (miRNAs) are a class of endogenous short, highly conserved and single-stranded non-coding RNA molecules, containing about 19-25 nucleotides, that regulates gene expression at the post-transcriptional level through binding to the 3′or 5′-untranslated region of target mRNA, leading to translational repression or mRNA degradation [5]. MiRNAs play essential roles in multiple biological processes, including cell maintenance, development, and immune responses. In addition, miRNAs participate in immune homeostasis, such as immune cell development, central and peripheral tolerance, and T helper cell differentiation [4, 6]. Dysregulated miRNA expression has been reported to be associated with diseases such as cancer, inflammation, cardiovascular diseases, autoimmune diseases (including MS), systemic lupus erythematosus, and rheumatoid arthritis [7–9]. For example, in *in vivo* studies, miR-30a down-regulated the expression of the interleukin-21 receptor, resulting in fewer Th17 cells, and alleviated experimental autoimmune encephalomyelitis (EAE) [10]. Wan et al reported that miR-30a directly targeted Traf3ip2 mRNA (coding Act1) and inhibited IL-17-mediated NF-κB and MAPK activation, leading to a decrease of inflammatory cytokines and chemokines [11]. O'Connell et al. demonstrated a role for miR-155 in regulating inflammatory responses, and miR155^(−/−)^ mice were highly EAE-resistant [12]. Mandolesi et al. used miR-142-3p^(−/−)^ mice to demonstrate that synaptic irregulars, which are modulated by IL-1β, and clinical and neuropathological manifestation of experimental autoimmune encephalomyelitis (EAE) disappeared [13]. Recent evidence suggested that miRNAs are highly stable in blood and are resistant to circulating ribonucleases. Therefore combined with its important roles in multiple biological processes, miRNAs in peripheral blood, are promising biomarkers for diagnosis and prognosis of MS. Moreover, miRNA mimics, inhibitors, and antisense oligonucleotides may become effective weapons in the treatment against MS [14, 15].

Numerous studies have focused on identifying MS-specific miRNAs through reliable methods such as “next-generation” sequencing, microarray analysis, and polymerase chain reaction (PCR), which resulted in generation of a myriad of data. However, a major issue with these achievements was that each experiment varied in the source and amount of sample and tissue of origin, leading to large differences in miRNA results, the correlation was low and some data rather controversial. The question remains, which miRNAs are MS-specific markers.

In this study, we isolated peripheral blood mono-nuclear cells (PBMC) from MS patients and healthy controls, and evaluated and compared their miRNA expression profiles, specific locations on human chromosomes. Using biological information methods, we constructed a network of these miRNAs and their susceptibility genes (including risk genes and miRNA target genes), and identified MS-related miRNAs. Furthermore, biological functions and pathways of MS susceptibility genes were thoroughly analyzed. The MS-related pathways included important pathways in which key protein kinases were encoded by susceptibility genes. Finally, our study focuses on miRNAs, susceptibility genes, expression profiles and their roles in MS. The results of our study may provide significant insight in the therapeutic treatment of MS.

## RESULTS

### Expression profile of MS-related miRNAs and locations on human chromosomes

Representative miRNAs were validated in a total of 321 MS patients and 333 healthy controls. A total of 21 differentially expressed miRNAs identified in PBMC of MS patients were validated in literature studies. Of the 21genes, 12 miRNAs were up-regulated, 7 miRNAs were down-regulated, and 2 miRNAs were controversial. In order to identify the specific location of the miRNA on chromosomes, miRNAs were mapped into the Gene Database (last Update: October 19, 2016). Interestingly, the miRNA cluster, miR-15a and miR-16-1, were both down-regulated and located at the same position on human chromosome 13 with different regions of 13q14.2. The roles of the two miRNAs in MS were similar, they both inhibited the apoptotic processes through modulating BCL2 gene expression which is highly expressed in CD4+T cells from relapsing-remitting (RR)-MS patients. Similarly, miR-140-5p and miR-328 were both down-regulated and located in close proximity on human chromosome 16 with different regions of 16q22.1. Guan et al. demonstrated that has-miR-140-5p may suppress encephalitogenic T helper type 1 (Th1) cell differentiation and that the expression level of this miRNA was inversely correlated with MS severity [16]. However, the role of miR-328 in MS remains unclear. We hypothesized that miR-328 and miR-140-5p may be part of a new miRNA cluster. Additionally, miR-125a (up-regulated) and miR-150 (expression controversial) were located on human chromosome 19q13.41, 19q13.33, respectively, however the roles of the two miRNAs in MS are not clear. Detailed information is summarized in Table 1.

**Table 1:** Overview of target miRNAs with expression and location on human chromosomes

miRNA	Expression	Patients/Controls	Location on human chromosomes	PMID
hsa-let-7d	Up	20/21;12/20	9q22.32	21621006
hsa-miR-744	UP	20/21;12/20	17p12	21621006
hsa-miR-93	Up	20/21;12/20	7q22.1	21621006
hsa-miR-26a	Up	20/20	3p22.2	24792898
hsa-miR-326	Up	20/20; 36/32	11q13.4	24792898 21949733
hsa-miR-21	Up	29/19	17q23.1	21875645
hsa-miR-146a	Up	29/19;36/32	5q33.3	21875645 21949733 21875645
hsa-miR-15a	Down	15/15	13q14.2 (50,049,119..50,049,201)83bp	22463747
hsa-miR-16-1	Down	15/15	13q14.2 (50,048,973..50,049,061)89bp	22463747
hsa-miR-140-5p	Down	22/22	16q22.1 (69,933,081..69,933,180)100bp	26780721
hsa-miR-155	Up?	36/32;10/10	21q21.3 (25,573,980..25,574,044) 65bp	21875645 22272099 21949733
hsa-miR-142-3p	Up	36/32	17q22	21949733
hsa-miR-145	Up	20/19;12/20	5q32	26780721 23731539
hsa-miR-146b	Up	40/40;29/19	10q24.32	21875645 24217794
hsa-miR-200c	Up	40/40	12p13.31	24217794
hsa-miR-125a	Up	40/40	19q13.41	24217794
hsa-miR-328	Down	40/40	16q22.1 (67,202,321..67,202,395) 75bp	24217794
hsa-miR-152	Down	40/40	17q21.32	24217794
hsa-miR-199a	Down	40/40	19p13.2	24217794
hsa-let-7g	Down	19/14	3p21.2	22108567
hsa-miR-150	Down?	19/14;29/19	19q13.33	21875645 22108567

### Relationship between miRNAs and susceptibility genes

**Figure 1:**
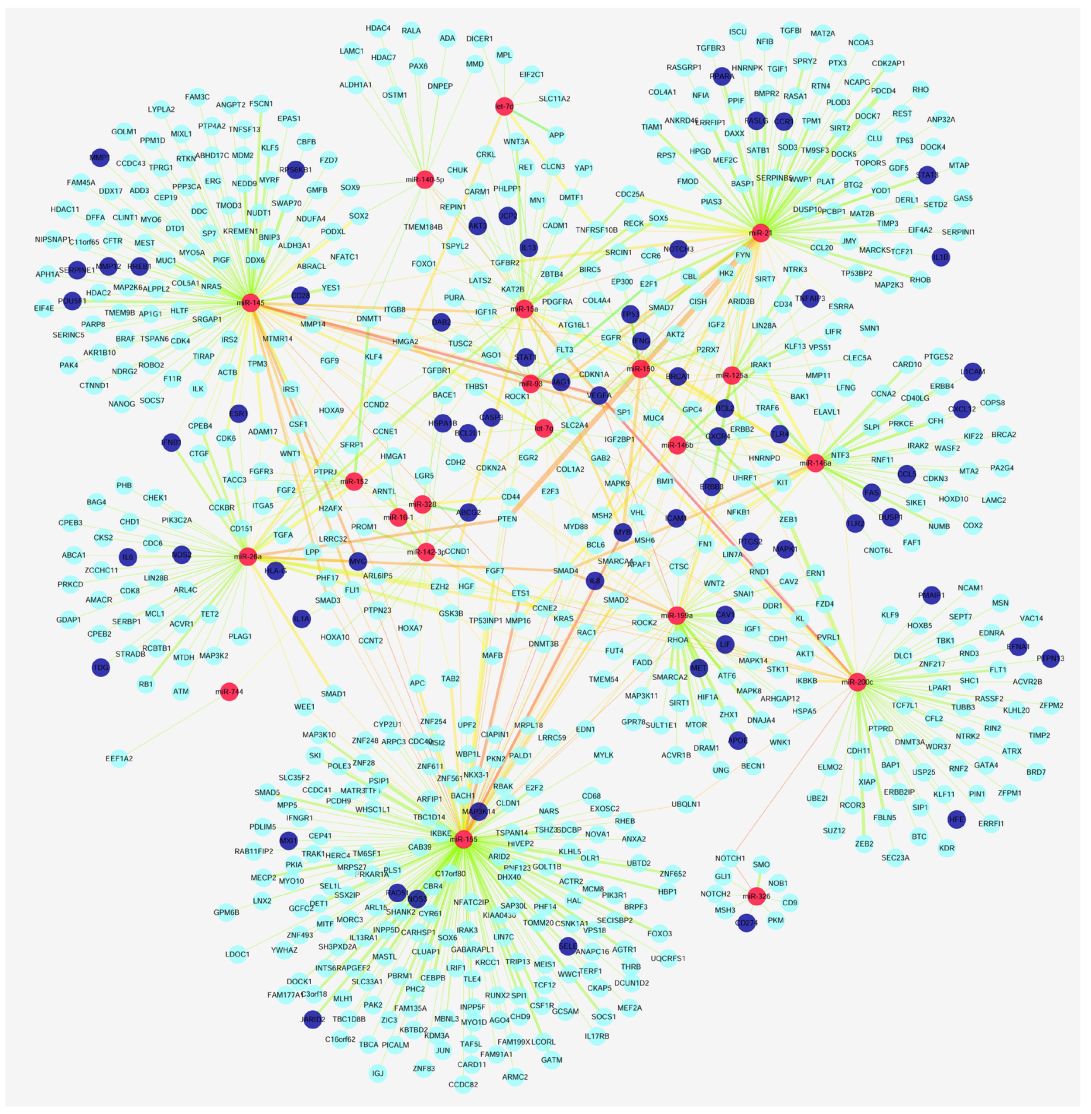
Relationship between miRNAs and susceptibility genes Red nodes represent miRNAs, dark blue nodes represent target genes that repeat the risk gene, and light blue nodes represent target genes. The larger map edge size represents target genes that were verified by an increased number of experiments, and the map edge of an increased red color highlights a close relationship between the nodes.

**Figure 2:**
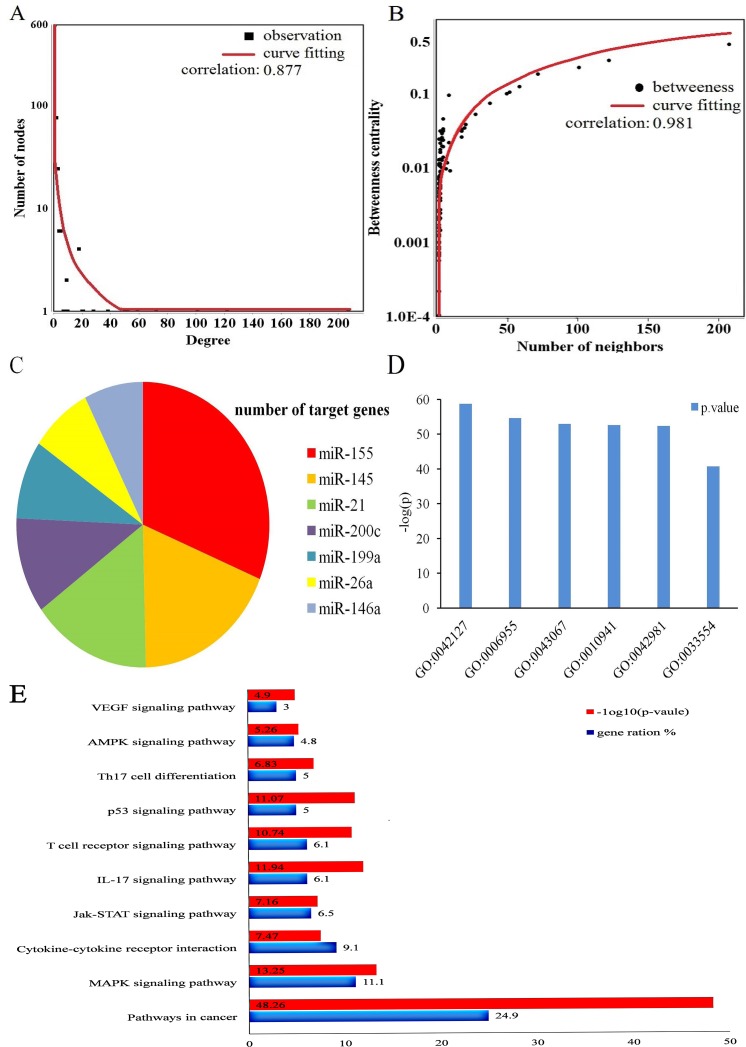
MiRNAs and their target genes biological function and topological properties **(A)** node degree distribution of miRNAs, susceptibility genes and curve fitting. **(B)** the node with more neighbors indicated the higher betweenness centrality, suggesting such nodes were more essential in MS. **(C)** the top 6 miRNAs with highest number of susceptibility genes respectively. **(D)** the significant function catalog of susceptibility genes through gene oncology (GO) analysis, and the p-value were log-processed. **(E)** The main pathways through susceptibility genes enrichment analysis, and the p-value were log-processed.

In this study, 1637 susceptibility genes were obtained including miRNA 710 target genes and 927 risk genes. Target genes from the miRTarBase Database were identified by experimental methods, such as luciferase reporter assay or green fluorescent protein reporter assay, ELISA, qRT-PCR, and Western blot analysis. Risk genes were taken from the OMIM database, GAD, and GWAS Central database. Target genes partially overlapped with risk genes, therefore Cytoscape [17] software was used to visualize the relationship between the genes. By matching target genes and risk genes, we observed that 69 of 710 target genes were repeated in risk genes. In the mapped 69 target genes, *VEGFA* was closely related with has-miR-145, miR-200c, miR-199a, and miR-21 compared to miR-140-5p, miR-15a, miR-125a, miR-93. *MYB* was more closely related with miR-155 compared to miR-150, miR-15a, and miR-200c. There was also a close relationship between *MYC*, miR-145, and miR-155. Of the 641 target genes, *SAMD4* was more significantly related with miR-155 compared to miR199a, miR-125a, miR-26a, and miR-146a. *PTEN*, which was confirmed as a target by most of the experiments, was more tightly related with miR-21 compared to miR-26a, miR-155 and miR-93. *ETS1* was more closely associated with miR-155 than with miR-200c and miR-145. *RHOA* was more related with miR-155 than miR-125a and miR-200c. *KRas* was significantly associated with miR-200c, miR-155, and miR152. *NOTCH1* was tightly associated with miR-200c and miR-326(detailed information is presented in Figure 1). All miRNAs and genes were closely correlated (Figure 2A and 2B). Among all 710 target genes, the maximum number of genes was derived from miR-155. The number of target genes decreased, for example from miR-145, miR-21, miR146a, miR-200c, miR199a, and miR-26a (Figure 2C).

### Identifying genes functional catalogues and MS risk pathways in MS

699 susceptibility genes were successfully converted in gene Entrez ID by Abosolute Gend ID Conversion Tools [18]. Then we used the cluster Profiler [19] package (statistical analysis and visualization of functional profiles for genes and gene clusters) in the R/Bioconducter to identify risk pathways in MS with a background of Homo sapiens. The cutoff criteria was P-value<0.01, minimal size of genes annotated by Ontology term for testing>10. The significant enrichment of biological processes contained GO:0042127:regulation of cell proliferation, GO:0006955:immune response, GO:0043067:regulation of programmed cell death, GO:0010941:regulation of cell death, GO:0042981:regulation of apoptosis, GO:0033554:cellular response to stress, which was in line with the current theories on MS pathology. Among the susceptibility genes, *VEGFA*, *ETS1*,*NOTCH1* were all involved in immune responses (Figure 2D).

“MS risk pathways” are associated with immune diseases or the immune system and highlight fundamental characteristics of autoimmune diseases. By using the cluster Profiler package, we identified 116 MS related pathways from Kyoto Encyclopedia of Genes and Genomes database (KEGG) database. The filter conditions: P-value<0.01, minimal size of genes annotated by Ontology term for testing>10. Based on the catalog of GO terms, the significant enrichment analysis of pathways included hsa05200(pathways in cancer), hsa04010(MAPK signaling pathway), hsa04060(Cytokine-cytokine receptor interaction), hsa04657(IL-17 signaling pathway), hsa04115(p53 signaling pathway), hsa04630 (Jak-STAT signaling pathway), hsa04660(T cell receptor signaling pathway), hsa04659(Th17 cell differentiation), hsa04152(AMPK signaling pathway), hsa04370(VEGF signaling pathway). These top 10 pathways are MS risk pathways (Table 2).

**Table 2:** Pathways associated with MS

KEGG Pathway	Count	P-value	adj.p-value
hsa05200 Pathways in cancer	115	5.46E-49	1.38E-46
hsa04010 MAPK signaling pathway	51	5.62E-14	4.29E-13
hsa04060 Cytokine-cytokine receptor interaction	42	3.4E-8	1.3E-7
hsa04630 Jak-STAT signaling pathway	30	6.9E-8	2.56E-7
hsa04657 IL-17 signaling pathway	28	1.15E-12	6.74E-12
hsa04660 T cell receptor signaling pathway	28	1.81E-11	9.69E-11
hsa04115 p53 signaling pathway	23	8.44E-12	4.73E-11
hsa04659 Th17 cell differentiation	23	1.48E-7	5.26E-7
hsa04152 AMPK signaling pathway	22	5.45E-6	1.53E-5
hsa04370 VEGF signaling pathway	14	1.26E-5	3.32E-5

### Hsa05200: potential hot pathway of MS

**Figure 3:**
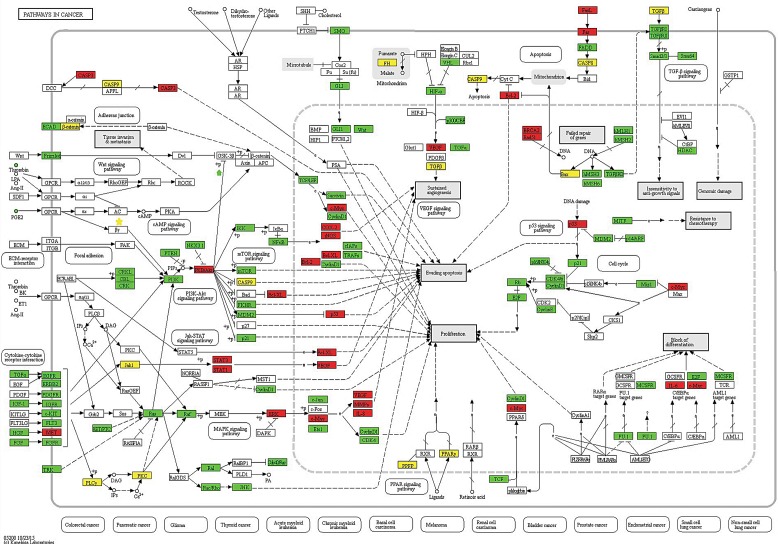
Schematic of the pathways in cancer from the KEGG database Proteins or complexes encoded by susceptibility genes are marked in white. A red background color represents target genes that relates with risk genes. A green background color represents target genes that do not relate with risk genes, and a yellow background color represents risk genes and their locations within the pathway.

In our study, Hsa05200 (pathway in cancer) was identified to be a most significant pathway of MS pathogenesis. Many genes in this pathway encoded cell surface receptors or important message signals such as risk genes: *ERK* (*MAPK1, MAPK3*), *MYC*, *VEGFA*, *MMP*, *IL8*, and target genes: *KRAS*, *RHOA*, *PTEN*, *SAMD4*, *IGF1R*, and *ETS1*. The specific locations of these genes have been marked in the pathway map (Figure 3). Proteins encoded by these genes regulate activation of sub-pathways including cytokine-cytokine receptor interaction, Jak-STAT signaling pathway, MAPK signaling pathway, and the TGF-β signaling pathway. For example, the MAPK signal transduction pathway, plays key roles in cell proliferation and differentiation, cell growth and survival, neuronal function and the immune response [20]. Thus, promising roles of hsa05200 have been observed.

### KRAS, p38, and MKK3/6 in MAPK signaling

Hsa04010 (MAPK signaling pathway) consisted of follow pathways: JNK(c-Jun N-terminal kinase) MAPK pathway, p38 MAPK pathway, extracellular signal-regulated kinase (ERK) pathway. Each cascade was comprised by three core kinases (MAP3K, MAPKK, and MAPK) and additional upstream (MAP4K) and downstream (MAPKAPK) components. The signal was spread through sequential phosphorylation or activations of the sequential kinases, and ultimately resulted in phosphorylation of MAPK and MAPKAPK components to target regulatory proteins [21]. In the ERK cascade, Ras (known as MAPKKKs) was activated by extracellular growth factors, insulin and G-proteins by binding directly to the N-terminus of the Ras protein which modified its structure by phosphorylation. The active signal was transmitted to ERK(a member of MAPK), after phosphorylation of MEK1/2, which are members of MAPKK [22]. Finally, this signal activated ETS1, which delayed activation in inflammatory cells may promote demyelination in Theiler's murine encephalomyelitis (a mouse model of multiple sclerosis) [23]. Activation of the ERK cascade positively participated in cellular activities, such as cell differentiation, proliferation, neuronal flexibility, and apoptosis [21]. *ERK* was considered a risk gene, whereas *KRAS* was a target gene, not a risk gene.

**Figure 4:**
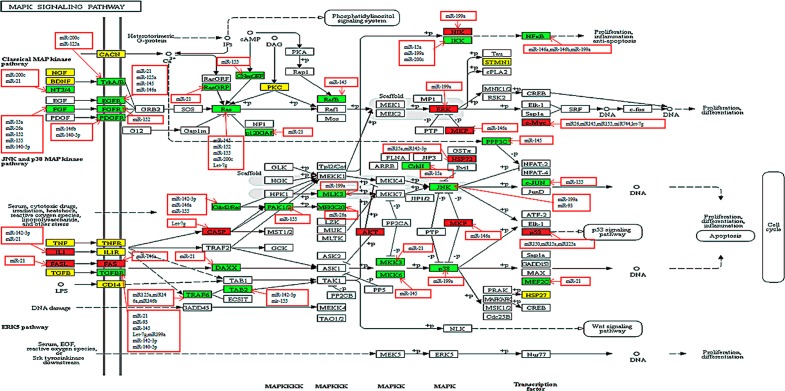
Relationship between miRNAs and susceptibility genes in the MAPK signaling pathway Proteins or complexes encoded by susceptibility genes are marked in a white background color. A red background color represents target genes that repeat with risk genes. A green background color represents target genes that do not repeat with risk genes, and a yellow background represents risk genes. MiRNAs with red frames negatively regulated their targets.

The p38 cascade can be activated by environmental factors, inflammatory cytokines, growth factors, insulin, transforming growth factor-α (TGF-α), ischemic stimulus. Activation of p38-related kinases required phosphorylation of two key MAPKKs namely, MKK3 (MAP2K3) and MKK6(MAP2K6) [20]. In this study, we discovered that *MAP2K3, MAP2K6*, and *P38(MAPK14)* were all target genes, and after activation of p38, several genes in the downstream pathway would be affected. This included *P53*, *ELK-1*, *MSK1/2*, and *MEF2C*. Among them, *P53* was a risk gene. Moreover, miR-199a negatively regulated the expression of the following genes: *JUN* (*MAPK9/10*), *p38* (*MAPK14*), *ERK* (*MAPK1*), *NFKB1*, and *NIK* (*MAP3K14*), therefore miR-199 primarily participated in the p38 signaling transmission process. Target genes of both miR-142-3p, and miR-21 encoded proteins that were mostly inflammatory factors, cell surface receptors or proteins located in signaling pathways upstream such as IL-1, TGFBR, TAB2, CD14, and MKK3 (MAP2K3). Therefore, miR-142-3p and miR-21 primarily regulated the activation process of the p38 the cascade (Figure 4).

### The roles of cytokine receptors in MS

Hsa04060 (cytokine-cytokine receptor interaction) is another pathway that has been reported to be associated with MS. This pathway contains chemokines, inflammatory cytokines, PDGF family, interferon family, IL-10 family, TNF family, IL-17 family, IL-1 family, and the TGF-β family. In our study, numerous genes encoded message signals or their receptors that referred to inflammatory cytokines. It is worth noting that several genes have been validated as risk genes, such as *IL2/IL2R*, *IL3/IL3R*, *IL4/IL4R*, *IL7/IL7R*, and *IL17/IL17R*. A large and growing body of evidence has investigated interleukins and found that their pathways played key roles in the development of MS. Especially, a strong relation has been shown between gene *IL7RA* (rs6897932), *IL2RA* (rs2104286), and the susceptibility, disability and development of MS [24, 25], indicating its importance in MS.

### Expression profile of pivotal susceptibility genes and relation with miRNAs

**Figure 5:**
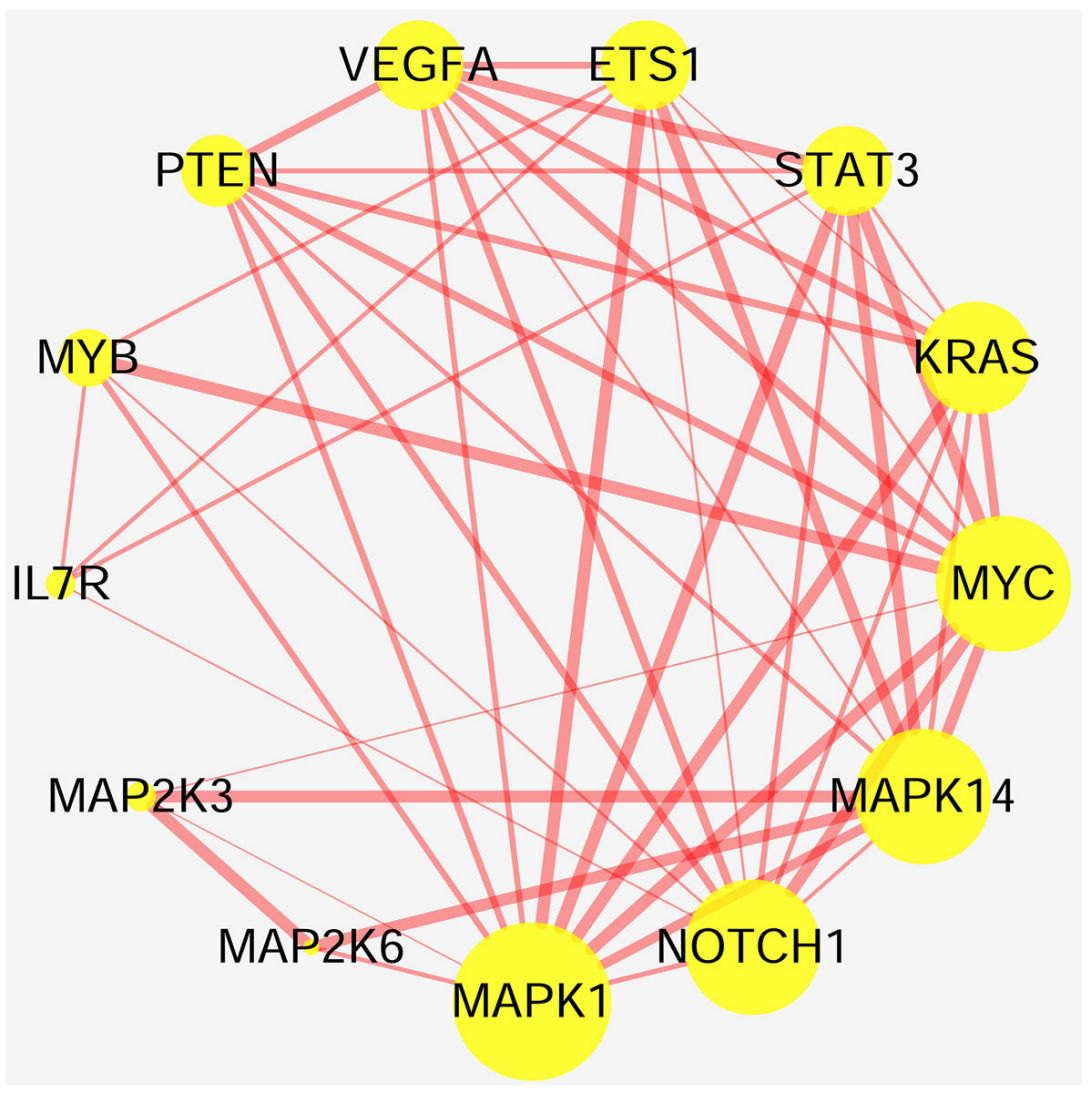
Relationship between highly susceptibility genes Nodes represent highly susceptibility genes and edges represent interaction between proteins. Nodes sizes are based on the number of degree: bigger nodes indicate larger degree. Size of edges represent combined scores: the more bold the edge, the higher the scores.

**Figure 6:**
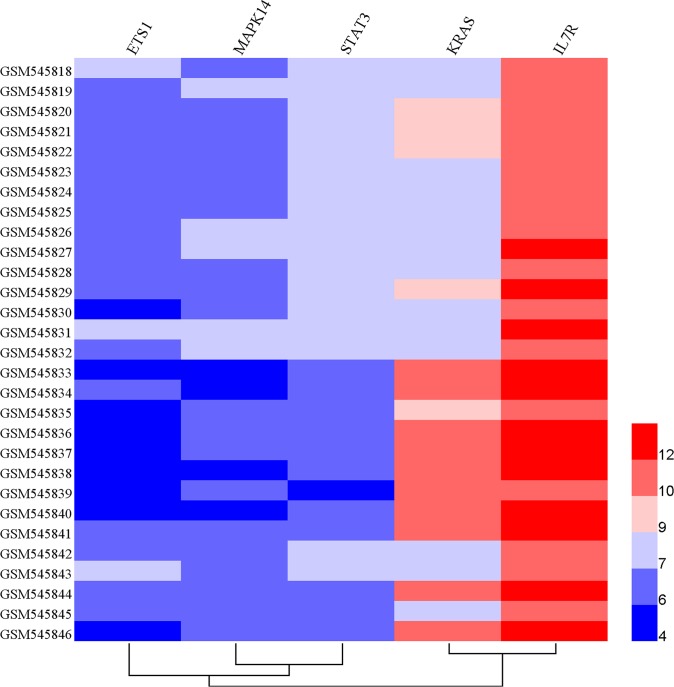
Hierarchical clustering analysis between 14 MS patients and 15 healthy controls based on differentially expressed genes Horizontal rows represent 5 genes, and columns represent 29 samples, including 15 samples derived from healthy controls (GSM545818-GSM545832) and 14 derived from MS patients (GSM545833-GSM545846). Red color represents over-expression, whereas blue represents under-expression.

To accurately and thoroughly identify pivotal susceptibility genes, we constructed a protein-protein interaction (PPI) network that was based on data from the STRING database. Seven nodes were exposed as hub genes (degree≥8, combined score≥0.9), including *ETS1, KRAS, MYC, MAPK1, MAPK14, NOTCH1*, and *STAT3* (Figure 5). Although *IL7R* was not properly matched it was demonstrated that IL7R was enriched on Th17 cells and could serve as marker of Th17 cells in experimental autoimmune encephalomyelitis (a common animal model of MS) [26]. Therefore, *IL7R* was considered another hub gene. Next, we evaluated the expression profiles of these genes exposed as hub genes in an independent genome-wide study of PBMC, GSE21942 [27] dataset from 15 healthy controls (Samples: GSM545818-GMS545832) and 14 MS patients (Samples: GSM545833-GSM545846), using the Gene Expression Omnibus Database. Five out of seven genes were differentially expressed between MS patients and healthy controls. The up-regulated genes included *IL7R*(log Fold Change=0.82, p<0.001) and *KRAS* (log Fold Change=1.31, p<0.001), while the down-regulated genes were *MAPK14* (log Fold Change= -0.70, p<0.001), *STAT3* (log Fold Change=-0.89, p<0.001) and *ETS1* (log Fold Change= -0.73, p<0.001) (Table 3, Figure 6). The expression of *MAPK1* and *NOTCH1* was not statistically significant. Interestingly, the interaction between pivotal susceptibility genes and characteristic miRNAs was determined using the miRSystem database. Hsa-miR-142-3p was a common target for *IL7R* and *KRAS*. In addition, *KRAS* was also the target of hsa-miR-155, hsa-miR-145, and hsa-miR-199a.

**Table 3:** Different expression level of highly susceptibility genes in healthy individuals versus MS patients

Gene Symbol	GSE21942 (14 MS patients vs.15 healthy controls)			
	ID	log FC	P-value	adj.p-value
*IL7R*	205798_at	0.82	<0.001	0.003
*KRAS*	214352_s_at	1.31	<0.001	<0.001
*MAPK14*	211087_x_at	-0.70	<0.001	<0.001
*STAT3*	225289_at	-0.89	<0.001	<0.001
*ETS1*	214447_at	-0.73	<0.001	0.043
*MAPK1*	212271_at	ns	ns	ns
*NOTCH1*	----	--	--	--

Shown are characteristic genes with a significant P.value and adjusted P-value (adj.p.value). Log FC (Log Fold Change) was logarithm2 of fold change; ns, not significant P.value.

## DISCUSSION

Although extensive research has been performed, effective therapy for the treatment of MS is still lacking. Prior studies have highlighted the importance of genetic susceptibility that highly correlated with the etiology of MS. Therefore, further studies are required to identify more effective therapies based on target genes. In this study, we focused on miRNAs from human PBMC and validated their expression levels. Through systemically and manually review of the literature, we identified 21 miRNAs that were experimentally validated. Despite the fact that expression levels of hsa-miR-150 and hsa-miR-155 were not consistent, previous studies have demonstrated a clear role for hsa-miR-155 in the pathological process of EAE, namely Th1 and Th17 cell differentiation. Hsa-miR-150 is involved in B cells, T cells, NK cell differentiation, and regulates the immune responses, however information on its roles in MS is limited [28–33]. Therefore, future studies on the role of hsa-miR-155 and hsa-miR-150 in MS are clearly warranted.

Hsa-miR-15a and hsa-miR-16-1 are both located on human chromosome 13q14.2 but in a different region. Their roles in MS were similar and both miRNAs regulated apoptotic processes of CD4+T cells by targeting the BCL2 gene [34]. Previous studies have demonstrated that compared with relapse patients, expression of the BCL2 gene was lower in MS remission patients, and was accompanied with lower levels of inflammation [35]. Hsa-miR-140-5p and hsa-miR-328 were both down-regulated and located at chromosome16q22.1 at different regions. In human PBMC of MS patients, a low expression level of hsa-miR-140-5p enhances disease severity and development of Th1 cells. During MS, hsa-miR-140-5p expression showed a greater reduction in the relapsing phase compared to the remitting phase, and inversely impacted the expression of IFN-γand STAT1 [16]. Although, the role of has-miR-328 in MS was not clear, in cervical cancer a low expression of hsa-miR-328 as well as hsa-miR-140-5p may be potential therapeutic targets by binding different functional target genes [36, 37]. Thus, we hypothesized that the mechanism of has-miR-328 in MS may be similar to that of has-miR-140-5p. On human chromosomes, the location of hsa-miR-125a and has-miR-150 was 19q13 with a different sub band. Both miRNAs played crucial roles in autoimmune diseases, such as lupus nephritis (LN) and myasthenia gravis (MG). Although the roles of the two miRNAs in MS were unclear, we suggested that the effects may be analogous, therefore future studies are essential.

Recent evidence suggested that MS may be related to abnormalities in apoptosis/cell death, microglia activation, blood-brain barrier functioning, immune responses, cytokine production, and oxidative stress. In MS, several cytokine-related signaling pathways as well as immune receptors have been discovered that were deregulated, including downstream molecules, such as JAK /STAT, NF-Kb, ERK1/3, P38 or JUN/FOS [38]. In our study, we used biological information to obtain 710 target genes (no duplicates), which covered 69 risk genes (approximately 10%). GO term and pathway enrichment analysis results were highly consistent with current theories of MS pathology described above.

It is well-known that T(H)17 lymphocytes, as a third subset of effector CD4 +T cells, were different from the classic lineages of Th1 and Th2 cells and played essential roles in the pathogenesis of MS [39]. *STAT3,* as a pivotal susceptibility gene, has been demonstrated a role in generating Th17 cell responses through CD4 in STAT3^−/−^ mice, therefore this gene was considered a marker of Th17 cells [40]. *ETS1* negatively regulated Th17 cell differentiation and negatively correlated with the severity of MS [41]. Lill et al. analyzed 21589 individuals (10796 MS cases and 10793 controls from Europe) through logistic regression and fixed-effect meta-analysis. *ETS1* (rs3809006, p=7.74×10(-9)) has a significant association with MS susceptibility [42]. *MAPK14* (*p38*) was found to positively regulate transcription of the *IL-17* gene and participated in the development of EAE through the induction of IL-17. Oral administration of a p38α (encoded by *MAPK14*) inhibitor obviously prohibited the progression of EAE and lowered the severity of neurological signs [43].

Based on the data described above, miRNA-based screening of MS-related genes seemed reliable. Such results provided the theoretical basis and guidance for the verification of future risk genes. In our study, the expression profile of *STAT3* in GSE21942 [27] was down-regulated in MS patients, whereas *IL7R* was up-regulated. The trend of two gene expression values was inconsistent because the gene expression data of GSE21942 were obtained from human PBMC in which Th17 cells were represented by only a small proportion. Therefore, we predicted that, as a MS biomarker, IL7R might be more reliable compared to *STAT3*. Previous studies have shown that *KRAS*, which encodes protein of the small GTPase superfamily, is involved in various malignancies, such as non-small cell lung cancer and colorectal cancer [44]. It could be used as a therapeutic approach for peritoneal mucinous malignancies by targeting the MEK- and PI3K-mediated pathway [45]. The role of *KRAS* in MS remains unclear, however through array analysis, *KRAS* expression levels were significantly up-regulated in MS patients. We concluded that *KRAS* may be a promising pivotal susceptibility gene.

Based on pathway enrichment analysis, hsa05200 was identified as a significant player in MS pathogenesis with a highest number of genes, which showed its promising role in MS. In hsa05200 pathway, MS-associated and risk genes were members of many vital sub-pathways, such as the MAPK signaling pathway, JAK-STAT signaling pathway, and cytokine-cytokine receptor interactions. For example, GWA studies demonstrated that the gene expression in the JAK-STAT signaling pathway significantly changed during MS [46]. The JAK-STAT pathway regulated immune responses through biological activities of interferon-gamma and granulocyte macrophage-colony stimulating factors [47]. Dysregulation of the JAK-STAT pathway resulted in autoimmune diseases, such as MS/EAE. Currently, many studies focus on the therapeutic targets of this pathway [47, 48].

The MAPK signaling pathway was a key signaling pathway that regulated a wide range of cellular processes, such as cell proliferation, differentiation, neuron function, and the immune response. During evolution, the pathway was one of the earliest signaling pathways to emerge and is highly evolutionarily conserved. This signaling pathway was possessed by all eukaryotic cells and is activated by environmental factors, inflammatory cytokines, growth factors, hormones, tumor necrosis factor, and ischemic injury [20]. If this pathway is dysregulated or functionally abnormal, it would lead to conditions, such as cancer, diabetes, autoimmune diseases, and developmental abnormalities [21]. P38 MAPK is a stress-induced kinase that plays an important role in inflammation. It is activated by a myriad of cellular stress and inflammatory responses. Activation of this sub-pathway released pro-inflammatory cytokines, such as IL10, IL-17 which are known to be involved in MS [49]. Di Mitri et al. demonstrated that the p38 MAPK pathway regulated Th17 cell differentiation, immune responses and participated in the pathogenesis of MS [50]. P38 inhibitors might be a new therapeutic tool to treat Th17-mediated autoimmune diseases [50], thus, our results were in line with previous studies.

MiRNAs, as crucial genetic negative regulators of gene expression, regulate various biological pathways [11]. Recent studies have shown that miRNAs in peripheral blood or the central nervous were potential biomarkers for diagnostic and prognostic use, therefore miRNA mimics/inhibitors may be potential therapeutic weapons to fight MS [15]. Target genes of miR-199a which were experimentally validated, encoded several vital proteins that are located at key positions in the MAPK signaling pathway, such as MAPK14 (p38), and JUN. In our study, we found that *KRAS*, a vital susceptibility gene, is a predictive target of miR-199a. During the initial phase of the study, miR-142-3p was considered a significant miRNA of MS. Mandolesi et al. found that in miR-142 knock-out mice IL1β-dependent synaptopathy as well as the clinical and neuropathological manifestations of EAE disappeared [13]. *In vivo* experiments indicated that miR-142-3p inhibition might delete IL1β-dependent synaptopathy in EAE animal model. Thus, Mandolesi et al. proposed that miR-142-3p was a key negative regulator of IL-1β-dependent synaptopathy in neuroinflammation [13]. By using the miRSystem, miR-142-3p was found a common predictive target of *IL7R* and *KRAS*. According to the relation between pivotal susceptibility genes and characteristic miRNAs, we hypothesized that miR-199a, miR-142-3p and their target genes (*IL7R, KRAS*) might act as MS therapeutic targets in the MAPK /JAK-STAT signaling transduction pathway.

In summary, through systemically scientific analysis, we identified that miR-155, miR-145, miR-21, miR146a, miR-200c, miR199a, and miR-26a are MS significant miRNAs, whereas miR-199a and miR-142-3p were characteristic miRNAs. We should deeply explore the clinical use of characteristic miRNAs and their potential target genes in MS. In addition, future studies are required to identify and validate MS-pivotal susceptibility genes (*KRAS*, *IL7R*) and their therapeutic roles in MS.

## MATERIALS AND METHODS

### Representative miRNA expression, location on human chromosomes

Characteristic target miRNAs were defined as those, which expression levels were significantly different between MS patient samples and healthy controls. In order to collect comprehensive information, “(“micrornas”[MeSH Terms] OR “micrornas”[All Fields] OR “mirna”[All Fields]) AND “multiple sclerosis”[All Fields] AND “humans”[MeSH Terms]” were used as key-words to search and review articles in the PubMed database (https://www.ncbi.nlm.nih.gov/pubmed). We read the literature of human species, which was published prior to October 30, 2016. Target miRNAs were obtained by following criteria: (1) each experiment contained at least 10 MS patients without any treatment and 10 controls. (2) experimental sample tissue must be from human PBMC. (3) target miRNAs were detected in an independent experiment both by microarray analysis and real-time –PCR (rt-PCR) with p<0.05 considered statistically significant. MiRNAs that met one of following criteria were also scored as targets:(i) miRNA emerged in an experiment in which only microarray was used, but was validated in an independent test of in which rt-PCR was used, p< 0.05. (ii) several special miRNA(s) from human PBMC have been confirmed in MS process in patients who were not treated. All target miRNAs were found at specific locations in the human chromosome with Gene Database (last Update: October 19, 2016) (https://www.ncbi.nlm.nih.gov/gene).

### Susceptibility genes: target genes of miRNAs and risk genes

Throughout this study, risk genes referred to genes that contained single-nucleotide polymorphisms (SNPs) associated with MS or genes that were experimentally validated to be involved in pathological process. The Online Mendelian Inheritance in Man (OMIM) database (Updated 21 October 2016) (http://omim.org/), Genetic Association Database (GAD) and GWAS Central database (release January 2016) (http://www.gwascentral.org/) were reviewed to obtain specific information on MS-related genes. Risk genes must be conform the following criteria: (1) the total number of patients or healthy participants enrolled in each study must be over 20. (2) biological methods of genes detected or validated should be reliable. (3) significant MS-associated SNP variants are identified by p<0.01. We obtained the related genes of these target miRNAs by reviewing the mirTarBase (Release 6.0: Sept. 15, 2015), a database storing experimentally confirmed microRNA-target interactions (http://miRTarBase.mbc.nctu.edu.tw/) [51–53]. The target genes must be conform the following criteria: (1) Luciferase reporter assay or green fluorescent protein reporter assay was used to validate the relation between miRNA target genes. (2) if experimental technology of reporter assay was not included, any two of ELISA, qRT-PCR or Western blot analysis must be used at the same time. (3) if ELISA, qRT-PCR and Western blot analysis was used, it must combined with biological method of the Luciferase reporter assay or green fluorescent protein reporter assay.

### Pathway, GO data and enrichment analysis

MS-related risk pathways were identified using the Kyoto Encyclopedia of Genes and Genomes (KEGG) database (updated October 1, 2016) and pathways were in line with the entries of the ‘immune system diseases of Human Diseases’ and ‘immune system of Organismal systems’. To identify MS risk, and which target and risk genes are involved in the MS pathway, we applied the cluster Profiler [19] package (statistical analysis and visualization of functional profiles for genes and gene clusters) in the R/Bioconducter. The cluster Profiler packages was used to enrich genes to GO functional categories and KEGG metabolic processes to discover biological function and a gene catalog. The cut-off criterion of the GO term was a Homo sapiens background, and p<0.01, minimal size of genes annotated by Ontology term for testing>10. The pathway criteria was a Homo sapiens background and p<0.01.

### Gene expression profile analysis and target miRNAs of pivotal genes

PPI networks were constructed based on important susceptibility genes. The network was evaluated based on two topological parameters, combined scores and degree. Degree≥8 and a combined score≥0.9 were cut-off criteria. Furthermore, Cytoscape [17] was used to construct and visualize the PPI network. Next, the expression profiling of GSE21942 [27] was downloaded from Gene Expression Omnbius (GEO, http://www.ncbi.nlm.nih.gov/geo/), which was based on the platform of GPL570: [HG-U133_Plus_2] Affymetrix Human Genome U133 Plus 2.0 Array. A total of 29 samples were enrolled in the database, including 14 MS patients (10 samples of PBMCs and 4 samples as a technical replicate) and 15 healthy controls. The differently expressed genes were identified by the limma [54] package (linear model for microarray data) in the R/Bioconducter. The cut-off criteria was p<0.05 and the log2FC (fold change)>0.6 or <-0.6 (fold change>1.5 or <0.6). Then, Heml [55] (Heatmap Illustrator, version 1.0) was used to create the heatmap and visualize the specific expression data of these important and pivotal genes. Moreover, the MiRSystem database, a integration of seven well-known miRNA target gene prediction programs: DIANA, miRanda, miRBridge, PicTar, PITA, rna22, and TargetScan (http://mirsystem.cgm.ntu.edu.tw/) [56] were used. According to the MiRSystem database, the interactions of characteristic miRNAs and pivotal genes were identified.

## Abbreviations

EAE: experimental autoimmune encephalomyelitis
ERK: extracellular signal-regulated kinase
FC: fold change
JNK: c-Jun N-terminal kinase
LN: lupus nephritis
MAPK: mitogen-activated protein kinase
MG: myasthenia gravis
miRNA: microRNA
MS: multiple sclerosis
PBMC: peripheral blood mono-nuclear cells
qRT-PCR: quantitative real time polymerase chain reaction
SNP: significant Single Nucleotide Polymorphisms
TGF-α: transforming growth factor-α
3′UTR: 3′-untranslated region
5′UTR: 5′-untranslated region.
